# Disseminated Gonococcal Disease Presenting As Achilles Tenosynovitis: Story of a Diagnostic Challenge

**DOI:** 10.7759/cureus.69654

**Published:** 2024-09-18

**Authors:** Rachel Lowe, Cynthia Nguyen, Jason Becker, Knogwan Yuenyongsagul, Erum Azhar, Abdul Waheed

**Affiliations:** 1 Department of Family Medicine, Dignity Health East Valley Family Medicine Residency Program, Gilbert, USA; 2 Department of Family Medicine, Creighton University School of Medicine, Phoenix, USA; 3 Department of Family Medicine, Dignity Health Medical Group, Arizona, Chandler, USA; 4 Obstetrics and Gynecology, Creighton University School of Medicine, Phoenix, USA; 5 Obstetrics and Gynecology, Dignity Health East Valley, Gilbert, USA; 6 Department of Family Medicine, Dignity Health Medical Group, Arizona, Gilbert, USA

**Keywords:** achilles tendonitis, ankle swelling, diagnostic medicine, disseminated gonococcal infection, infectious tenosynovitis

## Abstract

The Disseminated Gonococcal Disease (DGI) presents with varying signs and symptoms such as arthralgias and skin lesions to less commonly tenosynovitis posing a diagnostic challenge. In this case, a 64-year-old male presented to the emergency department with a 2-day history of flu-like symptoms, burning with urination, left ankle pain, erythema, and swelling. He met Systemic Inflammatory Response Syndrome (SIRS) criteria and was treated for presumed viral syndrome with supportive care. Blood cultures later grew *Neisseria gonorrhoeae*. He was called back to the hospital and treated with IV ceftriaxone and oral doxycycline. Further questioning following his treatment revealed a social history significant for recent unprotected receptive oral intercourse with a male partner. This case highlights the importance of early risk stratification and a higher index of suspicion in keeping DGI in the differential diagnosis of tenosynovitis with fever.

## Introduction

*Neisseria gonorrhoeae *(NG) is a Gram-negative diplococci that can be transmitted sexually (oral, genital, or anal) and perinatally. Among sexually transmitted infections, it ranks as the second most common cause worldwide [1]. A common presentation of NG usually stems from a genitourinary infection with symptoms of mucopurulent urethral discharge, dysuria, and possible pain and edema. However, NG infections can clinically present in a variety of ways spanning from an asymptomatic infection, genitourinary infection, pelvic inflammatory disease in females, and finally disseminated gonococcal infection affecting the skin, joints, gastrointestinal (GI) tract, etc [2].

Disseminated Gonococcal Infection (DGI) is a rare complication that results from the bacteremic spread of NG from its original site of inoculation. Between 2014 and 2019, only 0.06% of all gonococcal infections were found to progress to DGI across three surveillance areas in the United States [1]. However, the actual prevalence of this disease is likely higher due to the lack of surveillance. DGI was found to be most prevalent in men (64%) with ages ranging from 16-67 years old [3].

DGI typically follows two main presentations: arthritis-dermatitis syndrome or purulent gonococcal arthritis. Arthritis-dermatitis syndrome commonly presents with a triad of polyarthralgia, tenosynovitis, and dermatitis. The arthritis can be migratory and asymmetric in nature with the tendency to become purulent with simultaneous inflammation of several tendons. Lesions from dermatitis typically resolve within three to four days of onset. Purulent gonococcal arthritis can be mono- or oligoarticular, but commonly affects the knees, ankles, elbows, and wrists. Skin manifestations are typically absent in this presentation [4]. 

All patients suspected of DGI should undergo a nucleic acid amplification test and culture with antibiotic sensitivity. Patients with gonococcal arthritis can also have synovial fluid aspirated for analysis and gram stain. However, due to the intracellular nature of *N. gonorrhoeae*, Gram stain is only positive in <25-50% of cases [5]. Gram stains or aspirations that contain no organisms should not rule out NG infection. Treatment involves the administration of a third-generation cephalosporin; however, NG has been progressively developing resistance to antimicrobials commonly used for treatment over the past several decades. Previous studies have suggested that DGI is generally more susceptible to antimicrobials than uncomplicated gonococcal infections, but these estimates are based on older data [3].

Diagnosing DGI arthritis-dermatitis syndrome can pose a significant clinical challenge as it may present similarly to benign tendonitis or tenosynovitis, a common complaint in the primary care setting. Normal tendonitis is commonly treated with analgesics, rest, and physical therapy, while DGI arthritis-dermatitis requires the administration of third-generation cephalosporins. This report of DGI presents as Achilles tendonitis with foot and ankle swelling, which posed a diagnostic challenge both at ambulatory and initial emergency department visits.

## Case presentation

A 64-year-old male presented to the emergency department with a 2-day history of flu symptoms, burning with urination, and left ankle pain. His initial vitals from the emergency department showed a heart rate (HR) of 114 beats per minute, temperature of 37.8 C, blood pressure of 130/86 mmHg, saturation of 97% on room air, and respiratory rate of 18 breaths per minute. Initial labs from the emergency room were significant for a White Blood Cell count of 12,600 and lactic acid of 2.68 mmol/dL. The patient met Systemic Inflammatory Response System (SIRS) criteria based on his elevated heart rate and elevated white blood count greater than 12,000. Further workup included Protime/International Normalization Ratio (PT/INR), magnesium level, blood culture, lactic acid w/ reflux, urinalysis with reflex to culture, COVID/Flu/Respiratory Syncytial Virus, Complete Blood Count w/ differential, and Complete Metabolic Panel (CMP)** **as shown in Table 1. The viral panel was negative. Lactic acid was mildly elevated.

**Table 1:** Baseline labs at initial presentation to the emergency department

		Normal	Patient Values
CBC	WBC	4.2 - 10.2 10^3^/µL	12.6
	RBC	3.96 - 5.09 10^6^/µL	5.52 million/uL
	Hemoglobin	11.6 - 14.8	16.0 gm/dL
	Hematocrit	34.4 - 42.8%	46%
	Mean Corpuscular Volume (MCV)	77.8 - 93.2 fL	84.8 fL
	Mean Corpuscular Hemoglobin (MCH)	25.8 - 32.6 pg	29 pg
	Red cell Distribution Width (RDW)	11.6 - 14.5%	13.5%
	Platelets	141 - 464 1000/µL	189 thousand
Auto Differential	Neutrophils	38.3 - 74.8%	10.1 thousand/uL
	Immature Granulocytes	0.0 - 0.8%	na
	Monocytes	2.5 - 13.8%	1.1 thousand/uL
	Eosinophils	0.0 - 8.9%	0.2 thousand/uL
	Basophils	0.0 - 1.3%	0.1 thousand/uL
CMP	Sodium	137 - 145 mmol/L	137 mmol/L
	Potassium	3.5 - 5.1 mmol/L	3.9 mmol/L
	Chloride	98 - 107 mmol/L	100 mmol/L
	CO2	22.0 - 30.0 mmol/L	26 mmol/L
	Magnesium	1.6 - 2.3 mg/dL	1.8 mg/dL
Lactic Acid	Lactic Acid	<2.00 mmol/dl	2.68 mmol/dL

**Figure 1:**
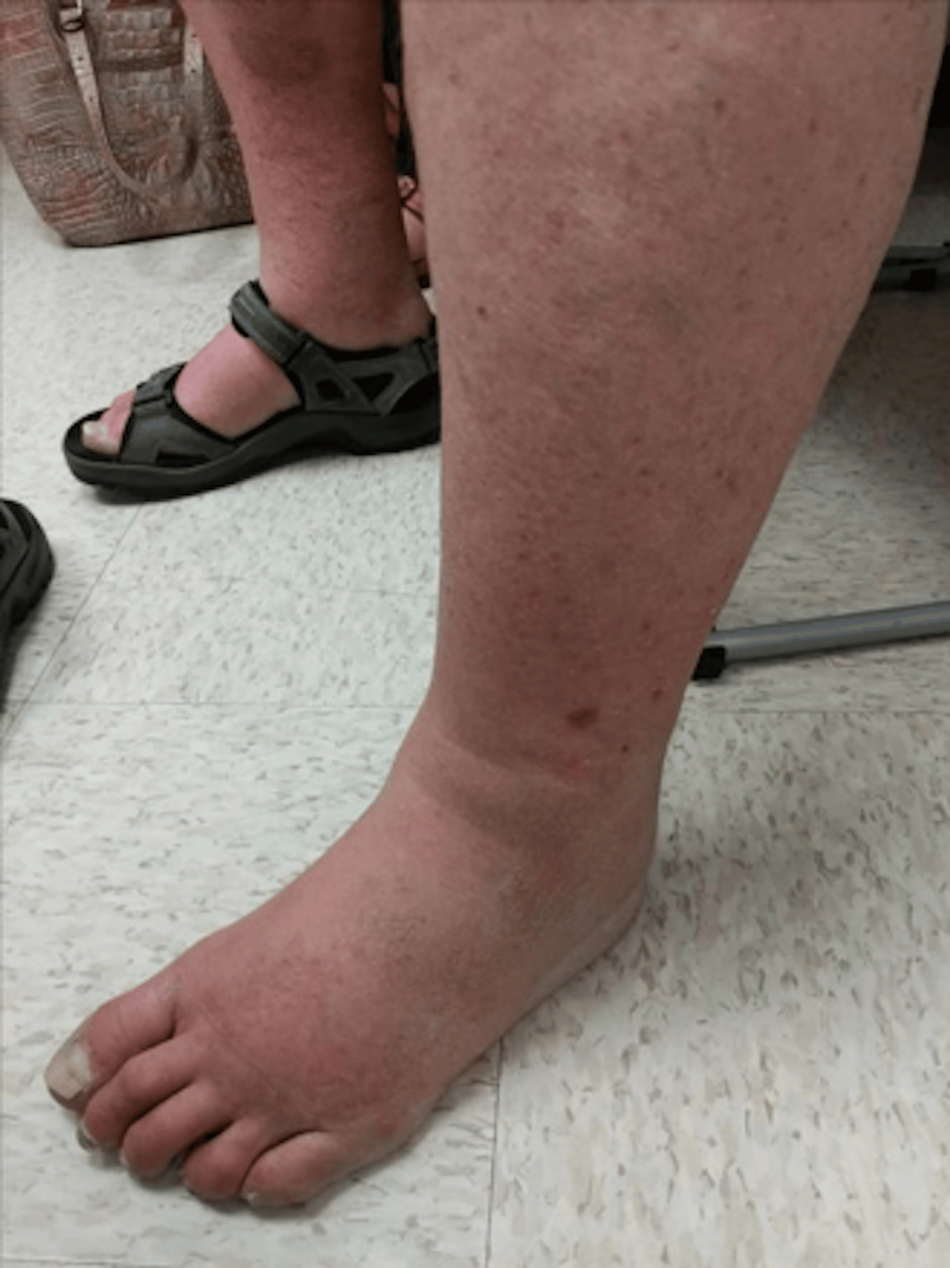
Photograph of remarkable swelling, erythema, and associated pain reported by the patient at presentation to clinic

The patient was recommended admission for a presumed viral illness, however, he declined and went home. The patient followed up with his primary care provider for concern of worsening left ankle pain, erythema, and swelling 5 days later. There was a concern for cellulitis vs Deep Venous Thrombosis (DVT),** **so a venous duplex Ultrasound (US) lower extremity** **was ordered and the patient was started on augmentin 875 mg PO BID. The swelling of the foot and ankle is shown in Figure 1. The venous duplex was negative for any DVT. The results of blood cultures from the Emergency Department were not available to the primary care provider's office by this point.

Two days after his initial emergency department visit the blood cultures grew Gram-negative cocci; multiple emergency department physicians made attempts over the following days to contact the patient. Blood cultures came back on day 10 following the initial ED visit growing *Neisseria gonorrhoeae*. The patient finally called back to the emergency department returning the provider's call regarding the results of the blood culture. At this time the patient was advised to come into the emergency department. He presented later that day. The patient presented with concern for mild left ankle pain with associated swelling. He was found to have tachycardia with a heart rate of 108 beats per minute, BP 110/74 mm Hg, respiratory rate of 15 breaths per minute, and lactic acid was elevated at 2.53 mmol/dL.

The patient was treated with intravenous (IV) ceftriaxone and admitted for treatment of disseminated gonococcal infection with associated tenosynovitis. During the course of his hospital admission, he continued to receive IV ceftriaxone and oral doxycycline 100 mg orally twice daily, which notably improved his symptoms. The patient was discharged home 3 days later with a Peripherally Inserted Central Catheter (PICC) line in place to continue IV antibiotics for 14 days. The patient followed up in his primary care clinic and noted resolution of ankle pain. At this time, he admitted to a history of unprotected receptive oral intercourse with a male partner. The patient has had no recurrence of symptoms and continues to follow up with primary care for the management of chronic disease.

## Discussion

This case of DGI presented with symptoms common to other common conditions including cellulitis, DVT, and acute inflammatory arthritis. The comprehensive workup in the emergency department including blood culture for fulfilling SIRS criteria later helped with making the accurate diagnosis. DGI commonly presents with monoarthritis or an asymmetric, severe polyarthralgia, which may be migratory or additive and usually peaks within a few days. Monoarticular involvement is more frequent than polyarthritis. Although any joint can be affected, the most common are the knees, wrists, and ankles. Gonococcal arthritis responds well to antibiotics when appropriate therapy is quickly initiated, but destructive arthritis may still be observed in immunocompromised patients [6]. DGI most commonly affects those 16-40 years of age, but it is important for physicians to recognize that DGI may affect many different age groups in addition to having a variety of presentations [7]. 

In this scenario, the patient presented with left ankle pain that continued to worsen and swell over the course of 5 days. There was no presence of characteristic gonorrhea petechial lesions. Positive blood cultures prompted the patient to return to the emergency department where he received IV ceftriaxone, the mainstay of gonococcal treatment. Another case report presenting with extensor tenosynovitis due to NG also received IV ceftriaxone for 2 weeks, in addition to a wash out of wrist flexor tendons. Washing the affected area with copious amounts of normal saline allowed for the clearance of bacteria and the potential mitigation of future bacterial destruction. While this is not the norm, washout may play a role in potential diagnosis and may be critical for recovery speed, especially in areas of intra-articular involvement where damage can be irreversible [8].

Diagnosis of DGI and prompt treatment is essential for symptom resolution and prevention of additional harm to the patient. Continued testing, incorrect diagnosis, and subsequent treatment may result in greater harm to the patient with chronic gonococcal infection potentially causing irreversible deformities [9]. Therefore, it is essential for physicians to obtain a thorough history and to keep an open mind to diagnosis to prevent long-term complications.

According to the guidelines, partners of affected individuals may be treated with a single 800 mg oral dose of cefixime, provided that concurrent chlamydial infection has been excluded. Otherwise, the partner may be treated with a single oral 800 mg cefixime dose plus oral doxycycline 100 mg twice daily for 7 days [10]. Some of the previous reports [11-13] have reported success with Point of Care (POC) ultrasound in aiding the diagnosis in the emergency department. Sonography has been shown to display fluid and possibly synovial thickening within the sheath of an infected tendon. Color Doppler can also provide some utility as increased flow can indicate infection, but this has been unreliable in differentiating infection in a joint and thus should not be the sole diagnostic evidence [12, 13]. Further studies delineating specific features of DGI on musculoskeletal ultrasound at the point of care both in the emergency department and ambulatory clinics are also recommended.

## Conclusions

Disseminated gonococcal disease is relatively uncommon but may present with symptoms easily confused with other common conditions. This case highlights that sexual history may aid clinicians raise the index of suspicion for disseminated gonococcal disease among those presenting with tendonitis, and tenosynovitis without obvious overuse syndromes or a history of trauma or cellulitis overlying joints.
